# Effect of Sulfate Carrier Addition on the Microstructure of Calcined Clay Blended Cements

**DOI:** 10.3390/ma18214972

**Published:** 2025-10-31

**Authors:** Maximilian Panzer, Sebastian Scherb, Nancy Beuntner, Karl-Christian Thienel

**Affiliations:** Institute for Construction Materials, University of the Bundeswehr Munich, Werner-Heisenberg-Weg 39, 85579 Neubiberg, Germany; sebastian.scherb@unibw.de (S.S.); nancy.beuntner@unibw.de (N.B.); christian.thienel@unibw.de (K.-C.T.)

**Keywords:** illitic clay, smectitic clay, kaolinitic clay, sulfate carrier addition, thermogravimetry, porosity, scanning electron microscopy

## Abstract

This publication builds on a previous paper proving the importance of sulfate carrier addition (sca) on the early compressive strength of calcined clay blended cements, especially when using a 1:1-dominated clay. This paper now aims to identify the background of these preceding findings at the microstructural level. For this purpose, a Portland cement is replaced by a calcined kaolinitic, smectitic and illitic clay to different levels at various sca. The microstructural investigations focus on hydrate phases, porosity and scanning electron microscopy of hardened pastes at 2 and 28 days. The microstructural properties of 2:1-dominated clay blends can be improved by the sca, but the benefit is small compared to 1:1-dominated clay blends. On the other hand, their portlandite content does not decrease as much, but can even increase slightly. At early hydration, the amount of bound water increases, and the porosity decreases with increasing sca for all blends. Neither the correlation of the compressive strength with the water binding nor with the porosity is high enough for a reasonable strength prediction using these two parameters.

## 1. Introduction

Calcined clays are forecast to account for 8% of cementitious material worldwide by 2050, although their share is currently negligible. With an estimated annual cement production of 5.1 billion tons, this corresponds to a demand of over 400 million tons of calcined clay per year by then [1]. To maximize the benefit from a calcined clay blended cement, its sulfate demand needs to be adjusted [2].

The type of calcined clay impacts its sulfate carrier adsorption [3] and thus affects the demand for sulfate carriers in the blend [4]. Also, the kinetics of alite hydration due to sulfate carrier addition (sca) differ depending on the type of calcined clay [5,6,7]. The most prominent clay minerals are kaolinite, illite, and smectite, which are present as dehydroxylated products after calcination (called metaclay). Their reactivity differs significantly depending on the mineralogical composition of the raw clays [8,9].

The R^3^ test can determine the reactivity of calcined clays [10,11]. Metakaolinite reacts significantly faster and releases more heat than metaillite or metasmectite [8,9,10]. This results from its higher amount of dissolved silicon and aluminum ions, as reported in studies on ion solubilities [8,12,13]. Especially the higher amount of aluminum ions due to its two-layer structure leads to a different behavior compared to metaillite and metasmectite [14].

In a cementitious system without sca, the high early reactivity of metakaolinite is equalized by the strong inhibition of alite hydration [5,7]. The use of metailllite and metasmectite reduces the alite hydration only slightly [5,6], so that—despite its own low reactivity—the same or higher early strengths of their blended cement can be achieved as with metakaolinite [9,15,16]. Scherb et al. [5] demonstrated that the sca to a metakaolinite blended cement increases early alite hydration. At 28 days, the effect of inhibition of the alite hydration fades so that the strength contribution of metakaolinite is higher than that of metasmectite and metaillite [9,13,15,16,17,18,19,20].

The preceding paper “The effect of sulfate carrier addition on the strength of calcined clay blended cements” [2] provides new knowledge about the strength contribution of various clay minerals during sca. Portland and Portland limestone cement were partially substituted by calcined 1:1-dominated and 2:1-dominated clays and by various amounts of sca. The following are the most important findings:

- The sca in the calcined clay blended cements delayed the early occurrence and reduced the high intensity of the aluminate peak. 1:1-dominated clays required larger amounts of sulfate carrier to adjust the heat flow than 2:1-dominated clays.
- The effect of sca on the mortar compressive strength was more pronounced at 2 days than at 28 days, particularly for 1:1-dominated clays. Therefore, the sca ensuring the highest strength at 2 days was regarded as optimal.
- For blends with 2:1-dominated clays, the Activity Index increased by up to 10% in absolute terms with optimal sca, while oversulfation could already occur with sca exceeding 1 wt%. In contrast, some 1:1-dominated clay blends did not reach oversulfation even with 9 wt% addition, and their Activity Indexes rose by up to 60% in absolute terms. Basically, illitic and smectitic clays had the same strength response to sca.

Investigations on the heat flow of calcined clay blended cements confirm the later occurrence of early and intense aluminate peaks and the loss of intensity resulting from the sca [3,4,5,21,22,23,24,25,26,27,28,29,30,31]. They also show that more sulfate carrier is required for blends with 1:1-dominated clays than with 2:1-dominated clays to achieve the same effect [4,5,27,28,31].

The sca ensures that 1:1-dominated clays provide the highest strength contribution at an early age [2]. But the strength gain due to sca was observed only until 3 days [21,22,23], since sulfate carrier essentially influences the formation of alite in early hydration [5]. Thus far, the impact of sca on the strength of 2:1-dominated clay blends has hardly been investigated. Apart from the preceding publication [2], only a smectitic clay was used, which had a high SO_3_ content, so that no sca was required in the blend [27,28].

Research on sca in calcined clay blended cements has focused on 1:1-dominated clays [3,21,22,23,24,25,26] and only rarely investigated 2:1-dominated clays [5,6,27,28]. This also applies to microstructural investigations, which were rarely examined with sca. The existing results are summarized in the following.

Without sca, cement replacements with metakaolinite increase the amount of bound water, which is greater at 2 days than at 28 days [22,32,33]. Metaillite and metasmectite also raise the water binding, but not as much as metakaolinite [5,13]. The lower pozzolanic reaction and the lower content of reactive aluminum of the 2:1 phyllosilicates are mentioned as reasons. The sca increases the amount of bound water in the 1:1- and 2:1-dominated calcined clay blends until 28 days due to ettringite formation [6,27]. In addition to the aluminate clinker reaction [34], the sulfates react with the released aluminum ions from the calcined clay to ettringite [2,35]. Calcium from the alite hydration is required, since C_3_A is not involved in this reaction [26,36]. The solubility of aluminum ions from metakaolinite is so high that the maximum possible theoretical ettringite content is formed, assuming all sulfates are used for ettringite formation [26]. There is no lack of aluminum even if the aluminate clinker reaction is completed and sulfate is still available.

Replacements with metakaolinite result in a lower portlandite content (related to the cement used), which becomes more pronounced with increasing hydration age, since its pozzolan reaction develops over time [3,21,22,25,26,32,33,37,38,39,40,41]. In comparison, 2:1-dominated clays do not have such a high portlandite consumption as their pozzolanicity is lower [5,13,16,28,42]. The sca in 1:1- and 2:1-dominated calcined clay blends further reduces the portlandite content [6,25,26,27]. The reason given is the consumption of portlandite for the formation of ettringite.

The porosity of the hardened pastes is increased and refined by replacements with metakaolinite until 28 days [13,21,26,38,43,44,45]. Later, a decrease in porosity is observed [13,37,41,46]. The time-dependent differences are due to the increasing degree of reaction of metakaolinite over time. In comparison, replacements with 2:1-dominated clays cause higher porosity because their pozzolanic reactivity is not as strong [13,18]. Mishra et al. [43] show a way to reduce the porosity of metakaolinite blends by sca. This is also possible with clays that contain less kaolinite but other clay minerals such as smectite and illite [35].

In scanning electron microscopy (SEM) images, a reduced ettringite formation by replacing Portland cement with calcined clay is evident [47]. When a sulfate carrier is added to 1:1-dominated calcined clay blends, an increased ettringite formation is visible in the SEM images [4,23,35,48]. The sca compensates for the sulfate adsorption of the calcined clay [3].

The study at hand aims to achieve a better microstructural understanding of the impact of the sulfate carrier on different calcined clay blends and explains the results of preceding strength tests in [2]. From a European perspective, it is necessary to investigate the interaction of sulfates with illite and smectite in more detail because they are the most abundant clay-size mineral groups in Europe [49]. In comparison to kaolinite, they have been neglected in scientific studies. For the first time, the necessity of sulfate adjusting binders depending on the type of clay mineral is demonstrated, as this is not currently known from calcined clays.

The paper focuses on the question: how does the sca influence the microstructural properties of cements blended with different calcined clay minerals?

Therefore, a Portland cement was mixed with calcined clays and sulfate carrier, resulting in 23 different binder compositions. A kaolinitic, a smectitic, and an illitic clay were used as 20 and 40 wt% replacing material. Depending on the type of calcined clay and replacement level, different levels of sca were chosen, which amounted to up to 9 wt%. On the hardened pastes, hydrate phases at 2 and 28 days were analyzed, as well as porosity and SEM images at 2 days.

## 2. Materials and Methods

### 2.1. Materials and Binder Compositions

The Portland cement CEM I 42.5 N, the calcined clays PP, Ill-E and Smk, and the sulfate carrier (anhydrite) from the preceding publication [2] were also used for the microstructural investigations as binder. Their mineralogical and chemical compositions and physical parameters, already given in [2], are repeated in Table A1, Table A2 and Table A3 for the ease of the reader. Statements on metakaolinite refer to PP (93 wt% kaolinite), on metaillite to Ill-E (67 wt% illite), and on metasmectite to Smk (54 wt% smectite). Their reactivity is indicated in Table 1 by the cumulative heat release in the R^3^ test [11] at 24 and 168 h (from [2]) and the solubility of aluminum and silicon ions after 20 h of elution in 10% NaOH solution [50]. The cumulative heat release of PP is consistently many times higher than for the 2:1-dominated clays, especially at an early age. The same applies to ion solubilities.

Table 2 provides an overview of the different binder compositions. Thermogravimetry was made at 2 and 28 days, porosity and SEM images only at 2 days. The clays were incorporated exclusively in calcined and ground form, replacing 20 or 40 wt% of the binder in the blended cement pastes.

Table 3 presents the relative binder compositions used for producing the blended cement pastes. They are the same as in the preceding publication [2] for the mortars. The water-to-binder ratio (0.50) is also identical. The amount of binder, which consists of cement, calcined clay, and sulfate carrier, is consistently normalized to 100 wt%. In the nomenclature, only the nominal replacement levels of 20 or 40 wt% are considered, even though the actual calcined clay content is slightly lower due to the sca.

### 2.2. Thermogravimetric Measurement

Thermogravimetric measurements were made on the hardened blended cement pastes at 2 and 28 days. The blended cement pastes, composed of 5 g binder and 2.5 g water, were stirred for one minute after the binder was mixed for 30 s. After 24 h of airtight dry storage at 25 °C, the hydrated blended cements were stored in distilled water in crushed form for a further 24 h and 27 days at 25 °C. The hydration was stopped with isopropanol. The samples were kept for several days in isopropanol until measurement, for which they were dried at 60 °C immediately beforehand. The same amount of sample, ground by hand in a mortar, was always weighed and analyzed in the Simultaneous Thermal Analyzer STA 449 F5 Jupiter (Netsch-Gerätebau GmbH, Selb, Germany) at a heating rate of 2 K/min in the temperature range from 25 to 900 °C. A sufficiently fine grinding was emphasized for all samples. The dehydration temperatures of the hydrate phases in the cement paste are taken from [39]. Dehydration up to 400 °C is divided into three stages: 25–140 °C (stage 1), 140–190 °C (stage 2), 190–400 °C (stage 3) [39]. Dehydration of ettringite and C-S-H phases takes place in stage 1, while that of monophases (AFm) as hemi- and monocarboaluminate, monosulfoaluminate, and C-A-S-H phases occurs in stages 2 and 3. The mass loss of the hardened paste in the individual stages (temperature ranges: T_1_–T_2_) is defined as H_2_O_T1–T2_ [wt%] and is called bound water. Portlandite (Ca(OH)_2_) dehydrates at approx. 450 °C. Using the tangent method of Marsh and Day [51], the individual temperature ranges of portlandite dehydration are determined (T_a_–T_b_). The portlandite content is calculated based on its dehydration (H_2_O_Ta–Tb_) according to Equation (1) and according to Equation (2) related to the proportion z of cement in the binder [52]. The ratio of the molar masses of portlandite M_Ca(OH)2_ and water M_H2O_ is 4.113, and the portlandite content refers to the sample mass at 400 °C.

(1) $${Ca(OH)}_{2}\;[wt\%]=\frac{M_{{Ca(OH)}_{2}}}{M_{H_{2}O}}\cdot \frac{H_{2}O_{T_{a}-T_{b}}}{1-\frac{{H_{2}O}_{25-400{}^{\circ}C}}{100wt\%}}=4.113\cdot \frac{H_{2}O_{T_{a}-T_{b}}}{1-\frac{{H_{2}O}_{25-400{}^{\circ}C}}{100wt\%}}$$

(2) $${Ca(OH)}_{2}\;[\frac{g}{100\;g\;cement}]=\frac{{Ca(OH)}_{2}\;[wt\%]}{z}$$

### 2.3. Porosity Measurement

The porosity of the hardened blended cement pastes at 2 days was determined using mercury pressure porosimetry according to DIN 66133 [53]. Except for grinding in mortar, the same samples were produced and prepared in the same way as those for the thermogravimetric measurements. After several days in isopropanol and subsequent drying at 60 °C, cubic pieces of the hardened pastes were measured in the Thermo Scientific Pascal Series mercury porosimeter (ThermoFisher Scientific, Germering, Germany) in the low-pressure unit (400 kPa) for pore radii down to 2 µm and in the high-pressure unit (400 MPa) for pore radii down to 2 nm. The porosity of the hydrated cement pastes is divided into four classes based on the pore size: >50 µm (air voids), 50—1 µm (capillary pores), 1—0.03 µm (microcapillary pores), and <0.03 µm (gel pores) [54]. Potential artifacts, such as the ink-bottle effect [55] and sample cracks [56], can occur in mercury pressure porosimetry, but are not taken into account in this series of measurements.

### 2.4. Scanning Electron Microscopy Images

Scanning electron microscopy (SEM) images of the hardened blended cement pastes at 2 days were taken on the samples that were already produced for the thermogravimetric measurements. After 24 h of airtight storage at 25 °C, the samples were crushed into small pieces and stored in air for a further 24 h. Flat pieces were analyzed in a Zeiss EVO LS 15 scanning electron microscope (Carl Zeiss Microscopy Deutschland GmbH, Oberkochen, Germany) with a LaB6 cathode and SE detector under high vacuum at a voltage of 20 kV after vapor deposition with gold.

### 2.5. Compressive Strength of Calcined Clay Blended Cements with Different Sca

The Activity Indexes (AI) of the blended cements from the preceding publication [2] are reproduced in Table A4. They are calculated by setting the compressive strength of the blended cement in relation to the reference cement (CEM I 42.5 N) at the same age. Also, the optimal sca is marked. Here, the highest 2-day Activity Index is the decisive criterion [2] and often results in the highest strength at 28 days as well.

## 3. Results and Discussion

### 3.1. Microstructural Investigations of Hardened Calcined Clay Blended Cement Pastes with Different Sca

The calcined clay blends have three variations of the sca: no sca (left column), medium sca (middle column) and highest sca (right column). The medium sca is the optimal one for blends with the 2:1-dominated clays (Table A4). For PP blends, the Activity Indexes with the optimal sca are similar to those with the highest (Table A4), so that the highest sca are considered optimal. The sca defined as optimal in this study are summarized in Table 4.

#### 3.1.1. Thermogravimetric Analysis of Hardened Pastes

**Figure 1:**
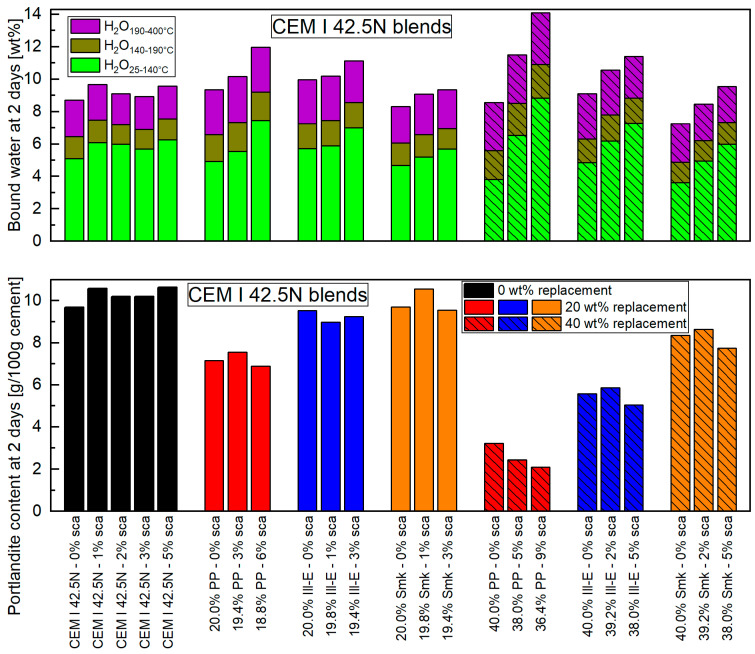
Bound water (**top**) and portlandite content (**bottom**) of different calcined clay blended CEM I 42.5 N at 2 days for 20 and 40 wt% replacements and with various sca. The samples with sole sca in the reference cement are included.

The bound water and portlandite content at 2 days are shown in Figure 1, with corresponding values listed in Supplementary Table S1. The amount of bound water is clearly higher up to 140 °C than from 140 to 400 °C for all samples. Without sca, Ill-E shows the highest and Smk the lowest bound water content at both replacement levels. The results are reasonable when considering the reaction degree of the different calcined clay minerals [5,6,13] and the inhibition of the alite hydration by metakaolinite [5,7]. Notably, the bound water amount of the AFm phases increased for all calcined clay blends compared to the reference cement, which is typical when using calcined clays [39]. While the sca in CEM I 42.5 N slightly changes the bound water amount, it significantly impacts calcined clay blends, especially up to 140 °C. The AFm phases’ amount remains nearly unchanged. The sca ensures that blended cements with PP and Ill-E achieve higher water binding than the reference cement, while those with Smk remain similar. A 9 wt% sca raises the bound water in the blend with 40 wt% PP by over 50%. The early excess of aluminum from PP (Table 1) results in the maximum ettringite content for their blends, as in [26,36]. A 9 wt% sca yields a calculated ettringite content of 21 wt% [2,34]. With the highest sca in 2:1-dominated clay blends (3 resp. 5 wt%), the ettringite formation does not halt due to insufficient aluminum release from 2:1-dominated clays (Table 1), as there is still enough C_3_A in the blend. At the same sca level, the bound water (stage 1) has increased equally across blends with the three different calcined clays compared to those without sca. Similar findings in [6,27,57,58] indicate that 1:1- and 2:1-dominated calcined clay blends bind more water with sca, primarily through ettringite formation.

At 2 days, only blends with 20 wt% Ill-E and Smk reach the portlandite content of the reference cement. A 20 wt% replacement by PP causes a decline of the portlandite content of more than 20%. The portlandite content drops significantly by increasing the calcined clay replacements from 20 to 40 wt%. This effect is most pronounced for PP with a 70% and lowest for Smk with a 20% decrease compared to the reference cement. The low portlandite content in the PP blends is due to the high early reactivity of metakaolinite [8,12,13]. The blends with Ill-E and Smk contain more portlandite, as the 2:1-dominated clays have a lower pozzolanicity. An increased consumption of portlandite by blends with 1:1-dominated clays compared to 2:1-dominated clays is also found in [5,27,28,42]. The sca has less impact on the portlandite content than the type of calcined clay or the replacement level. However, the highest sca ensures the lowest portlandite content. The added sulfates react with the released aluminum ions from the calcined clay and portlandite to ettringite [2,26,35], which forms more in PP blends due to the high amount of dissolved aluminum ions in metakaolinite (Table 1). Therefore, Silva et al. [27] found a decrease in portlandite content with sca in metakaolinite and metasmectite blends. This result is supported by [6,25,26].

The lower part of Figure 1 shows portlandite content relative to the amount of cement (Equation (2)). A calculation in relation to the total amount of binder (Equation (1)) better describes the passivation potential, which is responsible for the corrosion protection in the mortar and concrete. With such a relation, the portlandite content is reduced by the amount of replacement level. The corresponding values are given in Supplementary Table S1. PP stands out even more clearly, as its 40 wt% replacement does not even achieve 20% of the portlandite content of the reference cement.

**Figure 2:**
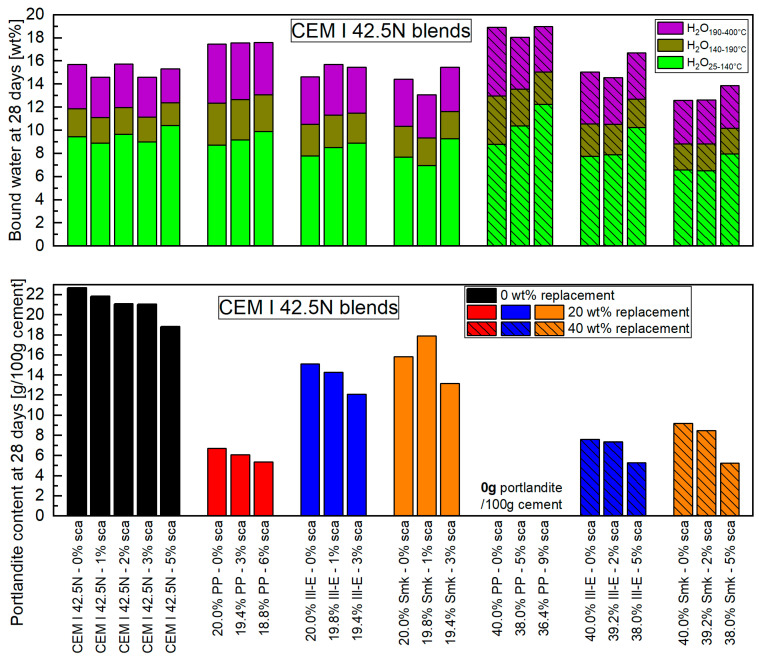
Bound water (**top**) and portlandite content (**bottom**) of different calcined clay blended CEM I 42.5 N at 28 days for 20 and 40 wt% replacements and with various sca. The samples with sole sca in the reference cement are included.

The bound water and portlandite content obtained at 28 days (Figure 2) are listed in Supplementary Table S2 and described in the following. The amount of bound water and the proportion of AFm phases are higher compared to 2 days. The total amount of water bound in PP blends exceeds that of the reference cement, at whose level 2:1-dominated clay blends are located. This is due to the high amount of dissolved silicon and aluminum ions of metakaolinite (Table 1). The impact of sca on the bound water at 28 days is not as high as at 2 days. The finding at 2 days that all blends have the highest water binding with the highest sca remains valid even at 28 days. The ettringite formed as a result of sca is still present. The amount of AFm phases in blends with PP decreases with rising sca. This applies in particular to the 40 wt% replacement by PP. The reason is the lack of portlandite (Figure 2(bottom)), which usually reacts with the abundant aluminum ions from PP to form AFm phases [26]. For 28-day-old metakaolinite blends, the literature agrees with us that the amount of bound water can be further increased by sca [27,32,33].

In course of clinker hydration, the portlandite content of the reference cement has more than doubled between 2 and 28 days. The type of calcined clay causes a difference in the portlandite content of their blends in this period. While it is reduced in PP blends, it increases in the 2:1-dominated clay blends, especially in the 20 wt% replacement. Hollanders et al. [42] found the same large difference in portlandite consumption of different calcined clays due to their pozzolanic reaction. Nevertheless, the portlandite content of the calcined clay blends stays below the reference cement at 28 days in this paper. With a 40 wt% replacement by PP, no portlandite could be detected in the blend. At the same replacement level, the portlandite content is approximately 70% lower for Ill-E and 60% for Smk compared to the reference cement. The high portlandite consumption at 28 days of metakaolinite is well documented in literature [21,22,32,33,38,40] and is also higher than that of metaillite and metasmectite [16,42]. As at 2 days, a sca lowers the portlandite content at 28 days in all blends. This is consistent with the observations of Silva et al. [27] for the metakaolinite blend but not for the metasmectite blend.

The portlandite content in Figure 2(bottom) refers to the cement and not to the binder. The values for latter are listed in Supplementary Table S2. In this case, blends with 20 wt% PP and 40 wt% Ill-E or Smk do not even attain 25% of the portlandite content of the reference cement.

#### 3.1.2. Porosity of Hardened Pastes

**Figure 3:**
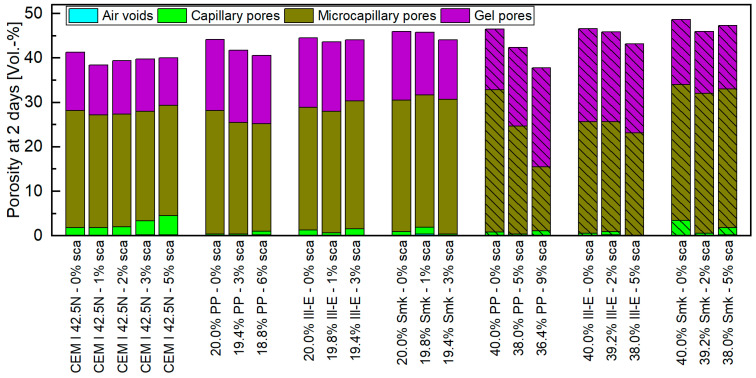
Porosity and its distribution of different calcined clay blended CEM I 42.5 N at 2 days for 20 and 40 wt% replacements and with various sca. The samples with sole sca in the reference cement are included.

The porosity and its distribution at 2 days are provided in Figure 3 and Supplementary Table S3. The amount of air voids and capillary pores is negligible in all samples. Microcapillary pores occur most often, followed by gel pores. The sole sca to CEM I 42.5 N hardly changes the porosity. It is approx. 40 vol.-%, and the ratio of gel to microcapillary pores is approx. 0.5. When using calcined clays without sca, this porosity is exceeded—especially with the 40 wt% blends. The fact that blends with Ill-E and Smk have a higher porosity than with PP is due to their significantly lower degree of reaction [5,6,13]. At 2 days, Zunino et al. [26] also reported an increase in porosity because of metakaolinite. Due to the pozzolanic reactions, metakaolinite reduces porosity with increasing age, so that it falls in these blends below that of the reference cement [13,37,41,46,59]. In comparison, blends with metasmectites and metaillites have a permanently higher porosity than with metakaolinites [13,18].

**Figure A1:**
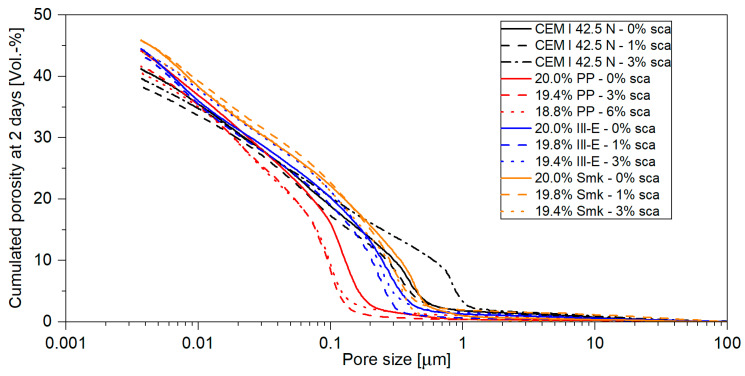
Cumulated porosity of the blends with 20 wt% replacement as a function of the pore size at 2 days. Samples with sole sca in the reference cement are included.

**Figure A2:**
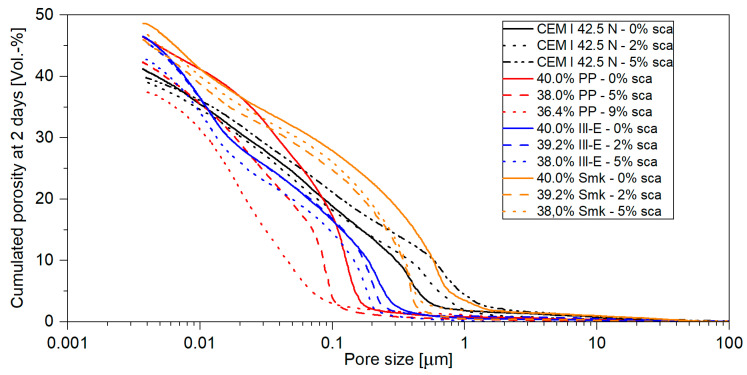
Cumulated porosity of the blends with 40 wt% replacement as a function of the pore size at 2 days. Samples with sole sca in the reference cement are included.

The porosity of the hardened calcined clay blended cement pastes decreases with sca. This positive effect can be attributed to the higher amount of hydrate phases (Figure 1(top)). Therefore, the porosity reduction is more pronounced for PP than for 2:1-dominated clays. The 9 wt% sca in a 40 wt% PP blended cement is the only sample in which the porosity of the reference cement is undercut. Thus, the porosity decreases by almost 9 vol.-% compared to its unsulfated blend. Its pore structure is significantly refined, which raises the ratio of gel to microcapillary pores from 0.43 to 1.55. This effect is less pronounced with a replacement by 20 wt% PP and does not occur if 2:1-dominated clays are used. The pore refinement of the PP blends is visible in Figure A1 and Figure A2. Pore refinements in favor of gel pores indicate increased C-S-H phases formation, as stated in [60], and is therefore congruent with the increase in alite hydration in metakaolinite blends [5]. The sca in a metakaolinite blend also reduces porosity in [43], but their measurement was only made at 28 days. The same applies to Lin et al. [35], who used a calcined clay with a low kaolinite content and other clay minerals (smectite, illite) and observed a pore structure refinement with sca.

#### 3.1.3. Scanning Electron Microscopy Images of Hardened Pastes

**Figure 4:**
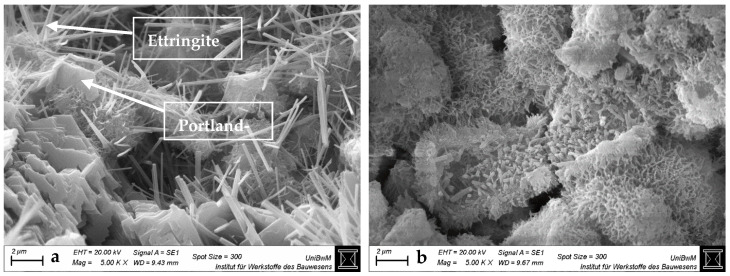
SEM images for hardened pastes with “CEM I 42.5 N—0% sca” (**a**) and “CEM I 42.5 N—5% sca” (**b**) at 2 days at a magnification of 5000.

**Figure 5:**
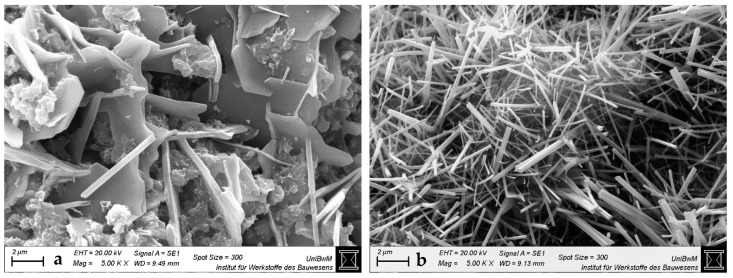
SEM images for hardened CEM I 42.5 N pastes with “40% PP—0% sca” (**a**) and “36.4% PP—9% sca” (**b**) at 2 days at a magnification of 5000.

**Figure 6:**
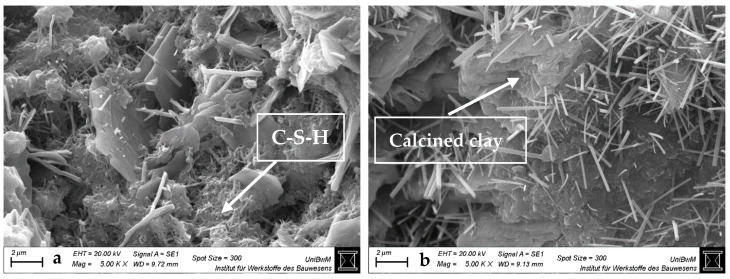
SEM images for hardened CEM I 42.5 N pastes with “40% Ill-E—0% sca” (**a**) and “38% Ill-E—5% sca” (**b**) at 2 days at a magnification of 5000.

**Figure 7:**
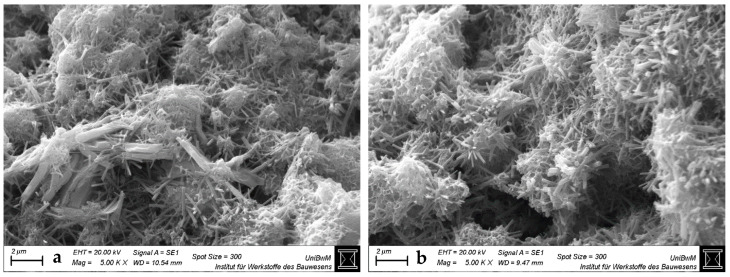
SEM images for hardened CEM I 42.5 N pastes with “40% Smk—0% sca” (**a**) and “38% Smk—5% sca” (**b**) at 2 days at a magnification of 5000.

**Figure A3:**
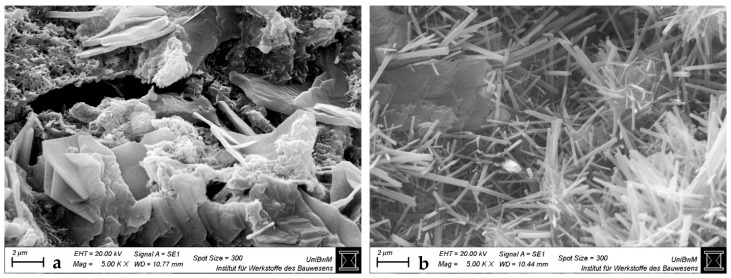
SEM images for hardened CEM I 42.5 N pastes with “20% PP—0% sca” (**a**) and “18.8% PP—6% sca” (**b**) at 2 days at a magnification of 5000.

**Figure A4:**
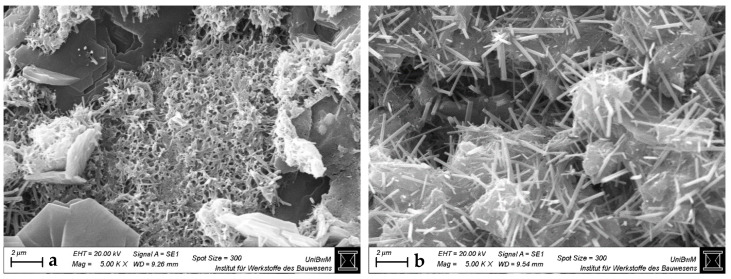
SEM images for hardened CEM I 42.5 N pastes with “20% Ill-E—0% sca” (**a**) and “19.4% Ill-E—3% sca” (**b**) at 2 days at a magnification of 5000.

**Figure A5:**
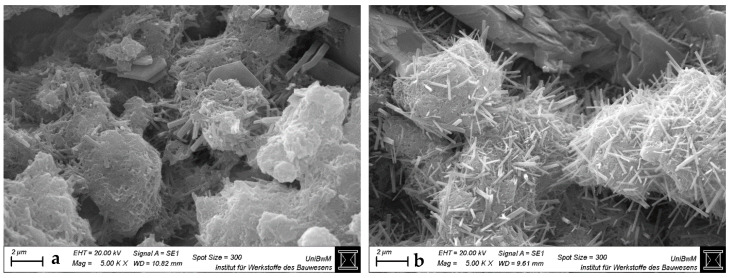
SEM images for hardened CEM I 42.5 N pastes with “20% Smk—0% sca” (**a**) and “19.4% Smk—3% sca” (**b**) at 2 days at a magnification of 5000.

Figure 4, Figure 5, Figure 6 and Figure 7 show the SEM images of the blends with 0 and 40 wt% clay replacement at 2 days without (a) and with highest (b) sca at a magnification of 5000. The images for the blends with 20 wt% replacement are provided in Figure A3, Figure A4 and Figure A5 and for the blends with medium sca in Supplementary Figures S1–S5. Important and often occurring hydration products are labeled by name at the points where they are best recognizable (Figure 4a and Figure 6). In pure cement pastes (Figure 4), fewer and shorter-fibered ettringite crystals can be seen due to the sca. This is in line with Hay et al. [23], who reported the same and attributed it to the refined pore network, which was also observed here (Figure 3). Replacements by calcined clays reduce the occurrence of ettringite significantly, which can be counteracted by sca. This effect is greater with 40 wt% replacements than with 20 wt%. Other publications also observed less ettringite due to the replacement by calcined clay in their SEM images and visually perceived the opposite with sca [4,23,35,47,48].

SEM images at 2 days are sufficient for statements on ettringite, because its formation is finalized. XRD analysis confirmed this for metakaolinite blends with very high sca [26]. Just as the preceding heat flow curves [2] did for the blends with the highest sca, which had their aluminate peaks not later than 2 days.

### 3.2. Correlation Between Microstructural Observations and Compressive Strength of Calcined Clay Blended Cements

#### 3.2.1. Bound Water of Blended Cements

**Figure 8:**
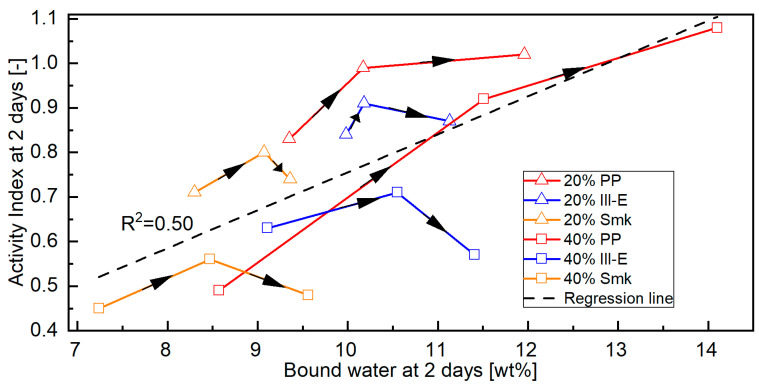
Activity Index of the CEM I 42.5 N blends with a replacement level of 20 and 40 wt% calcined clay at various sca (no, medium, highest) at 2 days as a function of their bound water content. The black arrows indicate the course of the curve with increasing sca.

Figure 8 compares the bound water content (Figure 1(top)) of blended cements at 2 days with their corresponding Activity Indexes (Table A4). A regression covering all calcined clay blends yields only a low correlation (R^2^ = 0.50) [61]. The Activity Index tends to increase as the amount of bound water increases. Separating the samples based on the type of calcined clay and the replacement level yields a clearer picture of the interaction between bound water and Activity Index due to sca. At 2 days, the amount of bound water of all calcined clay blends increases with increasing sca, while the Activity Index of blends with 2:1-dominated clays decreases beyond the medium (optimal) sca. The increasing ettringite formation seems to affect only the amount of bound water and no longer the Activity Index beyond the optimal sca. Thus, their curves in Figure 8 do not follow a linear trend, as observed in the PP blends, but decline at a certain point. The 2-day curves of the PP blends rise even beyond the medium sca, since the highest sca is the optimal one.

#### 3.2.2. Porosity of Blended Cements

**Figure 9:**
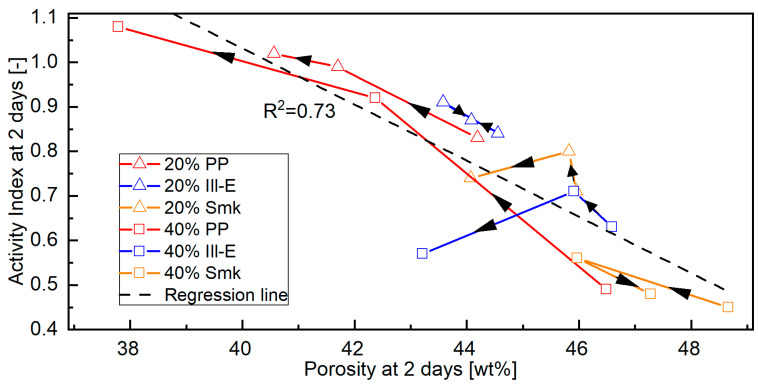
Activity Index of the CEM I 42.5 N blends with a replacement level of 20 and 40 wt% calcined clay at various sca (no, medium, highest) at 2 days as a function of their porosity. The black arrows indicate the course of the curve with increasing sca.

Figure 9 shows the Activity Indexes of the blended cements at 2 days (Table A4) as a function of their porosity (Figure 3). The regression covering all calcined clay blends is classified as high correlation (R^2^ = 0.73) [61]. Its course shows that the Activity Index increases as the porosity decreases. Such a process is caused by increased ettringite formation [39,62]. The porosity of all calcined clay blended cements decreases with increasing sca. The curves of the PP blends have a linear trend, because their Activity Index at 2 days increases up to the highest sca. In the blends with 2:1-dominated clays, the Activity Index at 2 days decreases beyond the middle sca, leading to a maximum in their curves at this point. The increased formation of ettringite at the highest sca reduces the porosity but no longer influences the strength. Lin et al. [35] reported the same effect at 28 days for a common clay that contained kaolinite, illite, and smectite as clay minerals. Antoni [63] mentioned a general trend between the two variables due to a lack of good correlation.

### 3.3. Effects of the Optimal Sca on Properties of Calcined Clay Blended Pastes and Mortars at Early Hydration

**Figure 10:**
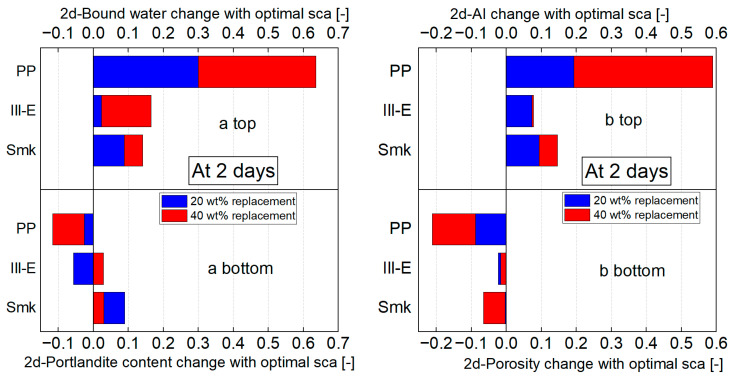
Change in bound water (**a top**), portlandite content (**a bottom**), porosity (**b bottom**) and Activity Index (**b top**) with optimal sca at 2 days of CEM I 42.5 N blends with 20 and 40 wt% calcined clays in relation to the reference cement. “change” refers to the respective blend without sca.

In this chapter, the changes in bound water, portlandite content, porosity, and Activity Index (AI) of the calcined clay blended cements due to optimal sca are compared (Figure 10). The focus is on 2 days, because the microstructural investigations and the previous compressive strength tests [2] revealed the stronger influence of sca at 2 days than at 28 days. The difference in values without and with optimal sca is divided by the value of the reference cement.

The optimal sca increases the amount of bound water at 2 days for all calcined clay blended cements (Figure 10(a top)). This effect is greatest with a replacement level of 40 wt% and with PP blends. This combination increases the amount of bound water at 2 days by over 60%. With 2:1-dominated clay blends, water binding increases by a maximum of 15%.

The choice of the optimal sca influences the portlandite content at 2 days (Figure 10(a bottom)). While PP blends lose up to 10% of the portlandite content of the reference cement, the opposite holds for blends with 20 wt% Smk that have an up to 10% higher portlandite content.

Figure 10(b bottom) shows a reduction in porosity at 2 days for all blends with the optimal sca. The reduction is particularly pronounced with PP blends, as the porosity loses more than 20% of the reference cement with a replacement level of 40 wt%. In comparison, blends with Ill-E do not even reach 5% and blends with Smk not even 10%.

Due to optimal sca, a PP blend increases its Activity Index at 2 days by approx. 20% with a replacement level of 20 wt% and by approx. 60% with a replacement level of 40 wt% (Figure 10(b top)). In contrast, the increase for blends with 2:1-dominated clays is around 10% at both replacement levels.

The optimal sca improves the compressive strength of the various calcined clay blends because their water binding increases and porosity decreases. The increased ettringite formation resulting from sca was identified as the cause. In addition, sca increases alite hydration in metakaolinite blends [5,7]. For this reason, the impact of the optimal sca is greater with the 1:1-dominated clay than with the 2:1-dominated clays. The optimal sca, which is defined on the basis of the highest strength, is 6 and 9 wt% for 1:1-dominated clay blends and 1 and 2 wt% for 2:1-dominated clay blends. Ettringite formation proceeds completely to the highest sca (3 and 5 wt%) in 2:1-dominated clay blends, but beyond the optimal sca (1 and 2 wt%), ettringite formation only affects their microstructure and no longer their strength. Due to the early release of aluminum ions from the metakaolinite (Table 1), the maximum possible ettringite content is reached in the 1:1-dominated clay blend at the highest sca (6 and 9 wt%), which improves the strength.

## 4. Conclusions

In this study, a sulfate carrier was added to calcined clay blended cements to evaluate its influence on their microstructure at early age. The following conclusions can be drawn:

- The impact of the sca on the microstructure is greater in blends with 1:1 than with 2:1-dominated clays. The higher the sca, the lower the porosity and the higher the amount of bound water in the calcined clay blends at 2 days. This is due to the increased ettringite formation, which is also most obvious in SEM images.
- Higher ettringite volume causes early strength increase in 1:1-dominated clay blends up to the highest sca used, but no longer in 2:1-dominated clay blends beyond a certain sca. Despite maximum water binding and minimum porosity at highest sca, their strength has decreased significantly.
- Another reason for the positive effect of sca on the microstructure and strength of 1:1-dominated clay blends is the increase of their alite hydration which is initially reduced by the high amount of aluminum ions from metakaolinite.
- The overall correlation between bound water and Activity Index of the blended cements at 2 days is moderate. The correlation between porosity and Activity Index is better, but still insufficient for a satisfactory prediction.
- The use of the optimal sca is particularly beneficial for early hydration. Blends with the 1:1-dominated clay benefit the most from improved properties (bound water, porosity, strength), but also have a significantly reduced portlandite content. With 2:1-dominated clays, the optimal sca improves the properties of their blends not as much, but the portlandite content is higher.

The microstructural investigations give a deeper insight into the results of the strength tests and improve the understanding of the impact of sulfate in calcined clay blends. This holds primarily for 2:1-dominated clays, as they have hardly been studied in contrast to kaolinitic clays. Further research is required to validate and broaden these findings using other clays. The effect of sca on the durability of calcined clay blended cements is worth looking at. The reduction of porosity indicates positive outcomes. An ongoing transfer project is analyzing the impact on concrete properties due to using a type II composite concrete additive (calcined clay—sulfate carrier—limestone powder). This will also provide important findings on the effect of sca in calcined clay blended cements in a field application.

## Figures and Tables

**Table 1:** Cumulative heat release in R^3^ test (from [2]) and aluminum (Al) and silicon (Si) ion solubilities of the calcined clays.

Material	Cumulative Heat Release [J/g Calcined Clay]		Ion Solubility in Alkaline Solution at 20 h [mmol/L]	
	24 h	168 h	Al	Si
PP	776	917	14.6	15.6
Ill-E	150	242	2.7	5.9
Smk	123	380	2.3	5.5

**Table 2:** Overview of the various binder compositions.

	Replacement	CEM I 42.5 N						PP										Ill-E						Smk					
Sca [wt%]		0	1	2	3	4	5	0	1	2	3	4	5	6	7	8	9	0	1	2	3	4	5	0	1	2	3	4	5
Microstructural investigations	0 wt%	X	X	X	X		X																						
	20 wt%							X			X			X				X	X		X			X	X		X		
	40 wt%							X					X				X	X		X			X	X		X			X

**Table 3:** Relative compositions of the binders for blended cement pastes.

	0 wt% Replacement	20 wt% Replacement		40 wt% Replacement	
Sca [wt%]	Cement [wt%]	Cement [wt%]	Calcined Clay [wt%]	Cement [wt%]	Calcined Clay [wt%]
0	100.0	80.0	20.0	60.0	40.0
1	99.0	79.2	19.8	59.4	39.6
2	98.0	78.4	19.6	58.8	39.2
3	97.0	77.6	19.4	58.2	38.8
5	95.0	76.0	19.0	57.0	38.0
6		75.2	18.8	56.4	37.6
9				54.6	36.4

**Table 4:** Overview of the different sca for the microstructural investigations of the calcined clay blended cements and their optimal sca in this study.

Variations of Sca for Microstr. Invest.	No Sca		Medium Sca		Highest Sca		Optimal Sca	
Replacement Level of Calcined Clay	20 wt%	40 wt%	20 wt%	40 wt%	20 wt%	40 wt%	20 wt%	40 wt%
PP blends	0		3	5	6	9	6	9
Ill-E blends/Smk blends	0		1	2	3	5	1	2

## Data Availability

The original contributions presented in this study are included in the article/Supplementary Material. Further inquiries can be directed to the corresponding author.

## Supplementary Materials

The following supporting information can be downloaded at: https://www.mdpi.com/article/10.3390/ma18214972/s1.

## Abbreviations

sca	Sulfate carrier addition.
SEM	Scanning electron microscopy.
AI	Activity Index.

## Appendix A

**Table A1:** Mineralogical and chemical composition and physical parameters of the cement (from [2]).

Mineralogical Composition [wt%]	CEM I 42.5 N	Chemical Composition [wt%]	CEM I 42.5 N	Physical Parameter	CEM I 42.5 N
C_3_S	60.4	SiO_2_	20.1	Particle density [g/cm^3^]	3.15
C_2_S	18.2	Al_2_O_3_	5.2	BET SSA [m^2^/g]	0.8
C_3_A	6.5	Fe_2_O_3_	2.8	d’ [µm]	31.9
C_4_AF	8.3	CaO	62.4	Water demand [%]	27
Calcite	0.8	MgO	1.5	Blaine SSA [cm^2^/g]	2742
Anhydrite	1.5	Na_2_O	1.5	f_2d_ [N/mm^2^]	19.2
Hemihydrate	0.7	K_2_O	1.9	f_28d_ [N/mm^2^]	54.9
Dihydrate	0.2	TiO_2_	0.1		
		SO_3_	2.8		
		LOI	1.8		

**Table A2:** Mineralogical composition and SO_3_ content of the raw clays (from [2]).

Raw Clay	Clay Group	Mineralogy [wt%]							SO_3_ [wt%]
		Kaolinite	Smectite	Smectite-Illite	Illite	Muscovite	Inert		
							Quartz	Others	
PP	1:1-dominated clay	93					5	2	0.1
Ill-E	2:1-dominated clay	10			67	7	12	4	0.2
Smk	2:1-dominated clay	7	54		4		17	18	0.1

**Table A3:** Physical parameters of the ground calcined clays and sulfate carrier (from [2]).

Material	Grain Size [µm]			BET SSA [m^2^/g]	Water Demand [%]	Particle Density [g/cm^3^]
	d_10_	d_50_	d_90_			
PP	1.0	4.8	39.2	18	57	2.61
Ill-E	1.9	15.8	54.6	71	53	2.71
Smk	1.4	14.4	60.6	38	45	2.71
Sulfate carrier	0.6	6.9	52.1	5	12	2.91

**Table A4:** Activity Indexes (AI) of 20 and 40 wt% calcined clays in CEM I 42.5 N with various sca at 2 and 28 days. The samples with sole sca in the reference cement are included. The optimal sca are marked.

0 wt% Replacement				20 wt% Replacement				40 wt% Replacement				
	Sca [wt%]	AI_2d_ [-]	AI_28d_ [-]		Sca [wt%]	AI_2d_ [-]	AI_28d_ [-]		Sca [wt%]	AI_2d_ [-]	AI_28d_ [-]	
CEM I 42.5 N	**0**	**1.00**	**1.00**									
	1	0.97	0.98									
	2	0.91	0.97									
	3	0.87	0.94									
	4	0.85	0.93									
	5	0.82	0.89									
				PP	0	0.83	1.25	PP	0	0.49	1.00	
					1	0.92	1.27		1	0.61	1.10	
					2	0.97	1.31		2	0.67	1.14	
					3	0.99	1.32		3	0.78	1.17	
					**4**	**1.02**	**1.35**		4	0.84	1.18	
					5	1.01	1.32		5	0.92	1.20	
					6	1.02	1.31		6	1.00	1.16	
									7	1.08	1.14	
									**8**	**1.09**	**1.11**	
									9	1.08	1.05	
				Ill-E	0	0.84	0.94	Ill-E	0	0.63	0.75	
					**1**	**0.91**	**1.01**		1	0.67	0.78	
					2	0.90	1.00		**2**	**0.71**	**0.81**	
					3	0.87	0.98		3	0.66	0.81	
									4	0.64	0.78	
									5	0.57	0.75	
				Smk	0	0.71	1.01	Smk	0	0.45	0.81	
					**1**	**0.80**	**1.08**		1	0.54	0.85	
					2	0.76	1.03		2	0.56	0.87	
					3	0.74	0.99		**3**	**0.57**	**0.85**	
									4	0.50	0.80	
									5	0.48	0.78	
