# Supplementary material for: Effect of Sulfate Carrier Addition on the Microstructure of Calcined Clay Blended Cements
Source: Materials (Basel). 2025 Oct 31;18(21):4972. doi: 10.3390/ma18214972 (PMC12608995; doi:10.3390/ma18214972)
Supplement: Supplementary file 1 [file materials-18-04972-s001.zip › materials-3803482-supplementary.pdf]

Supplementary Table S1. Bound water and portlandite content of different calcined clay blended CEM I 42.5 N at 2 days for 20 and 40 wt% replacements and with various sca. The samples with sole sca in the reference cement are included.

Calcined clay	sca [wt%]	$H_2O_{25-140°C}$ [wt%]	$H_2O_{140-190°C}$ [wt%]	$H_2O_{190-400°C}$ [wt%]	$H_2O_{25-400°C}$ [wt%]	$Ca(OH)_2$ [wt%]	$Ca(OH)_2$ [g/100g cement]
0 wt% (CEM I 42.5N)	0	5.11	1.36	2.23	8.70	9.69	9.69
	1	6.09	1.37	2.20	9.66	10.47	10.58
	2	5.98	1.21	1.91	9.10	10.00	10.20
	3	5.68	1.23	2.01	8.92	9.89	10.19
	5	6.27	1.28	2.02	9.57	10.10	10.63
20 wt% PP	0	4.92	1.66	2.77	9.35	5.72	7.15
	3	5.55	1.78	2.84	10.17	5.86	7.55
	6	7.45	1.74	2.77	11.96	5.19	6.90
20 wt% Ill-E	0	5.72	1.54	2.72	9.98	7.63	9.54
	1	5.90	1.55	2.73	10.18	7.12	8.99
	3	7.00	1.55	2.58	11.13	7.17	9.24
20 wt% Smk	0	4.68	1.39	2.23	8.30	7.76	9.70
	1	5.19	1.40	2.48	9.07	8.37	10.57
	3	5.69	1.27	2.40	9.36	7.42	9.56
40 wt% PP	0	3.81	1.79	2.97	8.57	1.93	3.22
	5	6.53	1.99	2.99	11.51	1.39	2.45
	9	8.82	2.09	3.19	14.1	1.15	2.10
40 wt% Ill-E	0	4.85	1.46	2.80	9.11	3.35	5.58
	2	6.19	1.61	2.75	10.55	3.45	5.86
	5	7.28	1.54	2.59	11.41	2.88	5.05
40 wt% Smk	0	3.61	1.27	2.36	7.24	5.01	8.35
	2	4.94	1.28	2.25	8.47	5.08	8.64
	5	6.00	1.32	2.24	9.56	4.41	7.74

Supplementary Table S2. Bound water and portlandite content of different calcined clay blended CEM I 42.5 N at 28 days for 20 and 40 wt% replacements and with various sca. The samples with sole sca in the reference cement are included.

Calcined clay	sca [wt%]	$H_2O_{25-140°C}$ [wt%]	$H_2O_{140-190°C}$ [wt%]	$H_2O_{190-400°C}$ [wt%]	$H_2O_{25-400°C}$ [wt%]	$Ca(OH)_2$ [wt%]	$Ca(OH)_2$ [g/100g cement]
0 wt% (CEM I 42.5N)	0	9.44	2.43	3.85	15.72	22.69	22.69
	1	8.90	2.20	3.50	14.60	21.62	21.84
	2	9.67	2.31	3.75	15.73	20.68	21.10
	3	9.01	2.14	3.44	14.59	20.42	21.05
	5	10.4	1.97	2.97	15.34	17.88	18.82
20 wt% PP	0	8.73	3.61	5.14	17.48	5.38	6.73
	3	9.17	3.48	4.91	17.56	4.74	6.11
	6	9.90	3.18	4.54	17.62	4.04	5.38
20 wt% Ill-E	0	7.79	2.72	4.12	14.63	12.09	15.12
	1	8.52	2.79	4.38	15.69	11.32	14.29
	3	8.90	2.58	3.98	15.46	9.39	12.10
20 wt% Smk	0	7.68	2.65	4.11	14.44	12.65	15.82
	1	6.96	2.38	3.72	13.06	14.19	17.92
	3	9.27	2.35	3.83	15.45	10.22	13.16

 https://doi.org/10.3390/ma18214972

40 wt% PP	0	8.79	4.20	5.92	18.91	0.00	0.00
	5	10.37	3.18	4.49	18.04	0.00	0.00
	9	12.25	2.81	3.94	19.00	0.00	0.00
40 wt% Ill-E	0	7.75	2.82	4.48	15.05	4.55	7.58
	2	7.89	2.63	4.04	14.56	4.33	7.37
	5	10.23	2.48	4.01	16.72	3.01	5.29
40 wt% Smk	0	6.57	2.27	3.77	12.61	5.51	9.18
	2	6.52	2.30	3.81	12.63	4.99	8.49
	5	7.97	2.22	3.67	13.86	3.01	5.28

Supplementary Table S3. Porosity and its distribution of different calcined clay blended CEM I 42.5 N at 2 days for 20 and 40 wt% replacements and with various sca. The samples with sole sca in the reference cement are included.

Calcined clay	sca [wt%]	Porosity [vol.-%]	Air voids [vol.-%]	Capillary pores [vol.-%]	Microcapillary pores [vol.-%]	Gel pores [vol.-%]	Ratio of gel to microcapillary pores [-]
0 wt% (CEM I 42.5N)	0	41.28	0.00	1.85	26.32	13.11	0.50
	1	38.36	0.15	1.66	25.40	11.15	0.44
	2	39.43	0.05	1.91	25.41	12.07	0.47
	3	39.76	0.22	3.10	24.65	11.79	0.48
	5	40.02	0.22	4.26	24.81	10.73	0.43
20 wt% PP	0	44.19	0.08	0.32	27.76	16.04	0.58
	3	41.70	0.05	0.33	25.09	16.23	0.65
	6	40.56	0.16	0.84	24.19	15.36	0.64
20 wt% Ill-E	0	44.55	0.15	1.14	27.59	15.67	0.57
	1	43.58	0.05	0.56	27.34	15.64	0.57
	3	44.08	0.12	1.42	28.81	13.73	0.48
20 wt% Smk	0	45.95	0.05	0.90	29.56	15.44	0.52
	1	45.82	0.34	1.59	29.77	14.12	0.47
	3	44.07	0.00	0.41	30.26	13.40	0.44
40 wt% PP	0	46.49	0.04	0.76	32.06	13.63	0.43
	5	42.37	0.02	0.40	24.27	17.68	0.73
	9	37.79	0.00	1.08	14.41	22.30	1.55
40 wt% Ill-E	0	46.59	0.09	0.50	25.09	20.91	0.83
	2	45.91	0.16	0.77	24.72	20.27	0.82
	5	43.21	0.00	0.15	22.94	20.13	0.88
40 wt% Smk	0	48.66	0.15	3.29	30.54	14.68	0.48
	2	45.97	0.02	0.50	31.51	13.94	0.44
	5	47.28	0.21	1.64	31.13	14.30	0.46

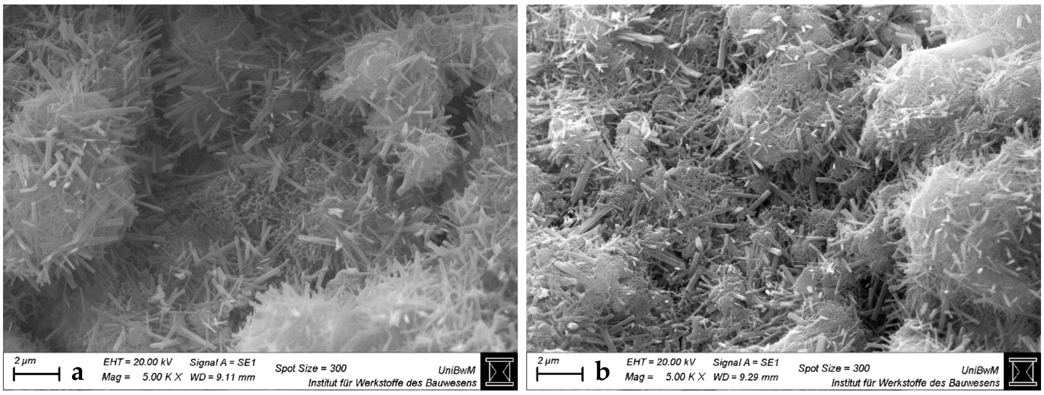

Supplementary Figure S1. SEM images for hardened pastes with "CEM I 42.5 N – 1% sca" (a) and "CEM I 42.5 N – 2% sca" (b) at 2 days at a magnification of 5,000.

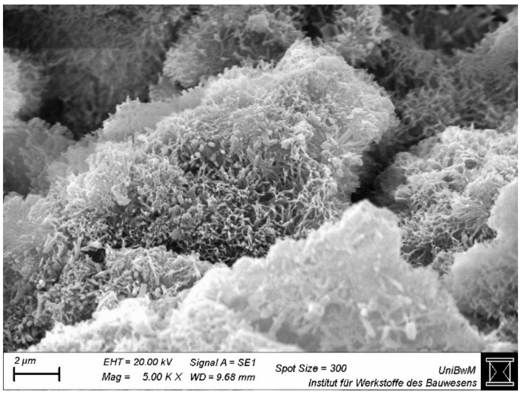

Supplementary Figure S2. SEM images for hardened pastes with "CEM I 42.5 N – 3% sca" at 2 days at a magnification of 5,000.

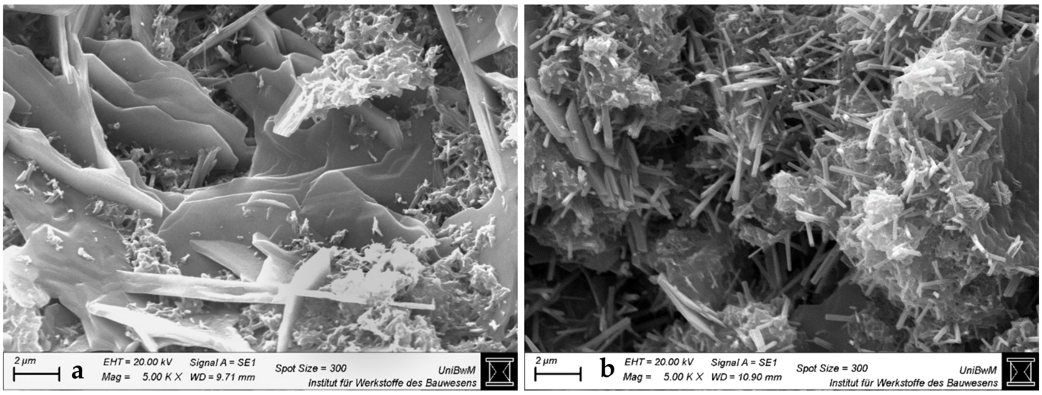

Supplementary Figure S3. SEM images for hardened pastes with "38% PP – 5% sca" (a) and "19.4% PP – 3% sca" (b) at 2 days at a magnification of 5,000.

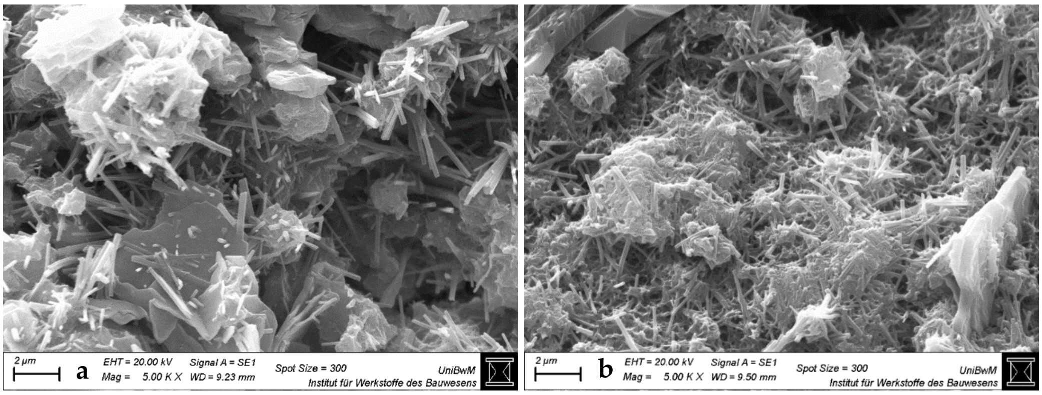

Supplementary Figure S4. SEM images for hardened pastes with “39.2% Ill-E – 2% sca” (a) and “19.8% Ill-E – 1% sca” (b) at 2 days at a magnification of 5,000.

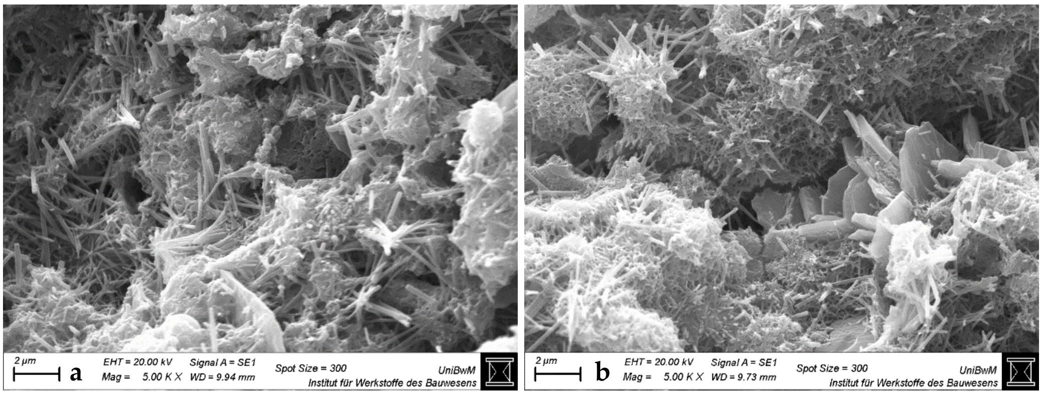

Supplementary Figure S5. SEM images for hardened pastes with “39,2% Smk – 2% sca” (a) and “19.8% Smk – 1% sca” (b) at 2 days at a magnification of 5,000.
